# Robotic Assistance System for Cone-Beam Computed Tomography-Guided Percutaneous Needle Placement

**DOI:** 10.1007/s00270-021-02938-7

**Published:** 2021-08-19

**Authors:** Michael Kostrzewa, Andreas Rothfuss, Torben Pätz, Markus Kühne, Stefan O. Schoenberg, Steffen J. Diehl, Jan Stallkamp, Nils Rathmann

**Affiliations:** 1 University Medical Center Mannheim, Department of Radiology and Nuclear Medicine, Medical Faculty Mannheim, University of Heidelberg, Theodor-Kutzer-Ufer 1-3, 68167 Mannheim, Germany; 2 Fraunhofer IPA, Fraunhofer Project Group for Automation in Medicine and Biotechnology, Medical Faculty Mannheim, University of Heidelberg, Theodor-Kutzer-Ufer 1-3, 68167 Mannheim, Germany; 3 Fraunhofer MEVIS–Institute for Digital Medicine, Am Fallturm 1, 28359 Bremen, Germany; 4 Department of Radiology, Kantonsspital Baden, Baden, Switzerland; 5 Mannheim Institute of Intelligent Systems in Medicine MIISM, Mannheim, Germany

**Keywords:** Leightweight robot, Cone-beam computed tomography, Image-guided needle placement, Robotic assistance system

## Abstract

**Purpose:**

The study aimed to evaluate a new robotic assistance system (RAS) for needle placement in combination with a multi-axis C-arm angiography system for cone-beam computed tomography (CBCT) in a phantom setting.

**Materials and Methods:**

The RAS consisted of a tool holder, dedicated planning software, and a mobile platform with a lightweight robotic arm to enable image-guided needle placement in conjunction with CBCT imaging. A CBCT scan of the phantom was performed to calibrate the robotic arm in the scan volume and to plan the different needle trajectories. The trajectory data were sent to the robot, which then positioned the tool holder along the trajectory. A 19G needle was then manually inserted into the phantom. During the control CBCT scan, the exact needle position was evaluated and any possible deviation from the target lesion measured.

**Results:**

In total, 16 needle insertions targeting eight in- and out-of-plane sites were performed. Mean angular deviation from planned trajectory to actual needle trajectory was 1.12°. Mean deviation from target point and actual needle tip position was 2.74 mm, and mean deviation depth from the target lesion to the actual needle tip position was 2.14 mm. Mean time for needle placement was 361 s. Only differences in time required for needle placement between in- and out-of-plane trajectories (337 s vs. 380 s) were statistically significant (*p* = 0.0214).

**Conclusion:**

Using this RAS for image-guided percutaneous needle placement with CBCT was precise and efficient in the phantom setting.

## Introduction

Minimally invasive, percutaneous, needle-based interventions are being performed more and more frequently in clinical routine. In addition to ultrasound, computed tomography (CT) is routinely used to place abscess drainages and for percutaneous tissue biopsies. Further minimally invasive tumor treatments, such as radiofrequency (RF) or microwave (MW) ablation as well as irreversible electroporation (IRE), are being requested with increasing frequency and require precise and efficient placement of needles in target lesions, which are often difficult to reach [1–3]. CT-guided interventions (CTGI) can be limited by gantry size and limited possibilities of gantry angulation, hence, requiring out-of-plane interventions, and more time for more complex procedures [4]. In addition, CTGIs can lead to increased radiation exposure for the medical staff and/or patient, depending on different factors, such as increased complexity of the intervention, the experience of the radiologists performing the procedure, and out-of-plane trajectories [3, 5]. With the increasing complexity and frequency of CTGIs, assistance systems might be helpful to facilitate more complex multi-angulated needle trajectories, allow for precise and safe needle placement, and decrease interventional time and radiation exposure [6].

Currently, several solutions are commercially available. These therapy support systems can be divided into active and passive systems as well as systems with and without imaging support and planning software [7]. Passive systems are positioned manually by the user. An example of such a passive system is the “SeeStar” (Apriomed, Uppsala, Sweden) [8]. It is a patient mounting system; here, the needle is held in place and its orientation locked before inserting the needle into the patient. Active systems are actuated by drives and typically include a planning software, for example, MAXIO (Perfint Healthcare, India) or Zerobot (Medicalnet Okayama, Japan) [5, 9].

The objective of this study was to evaluate a new, active robotic assistance system (RAS) consisting of a mobile platform in combination with a commercially available industrial lightweight robot (LWR) and including a dedicated planning software to enable percutaneous, image-guided needle placement in conjunction with a multi-axis C-arm angiography system for cone beam (CBCT) in a phantom setting.

## Materials and Methods

### Tools

**Fig. 1:**
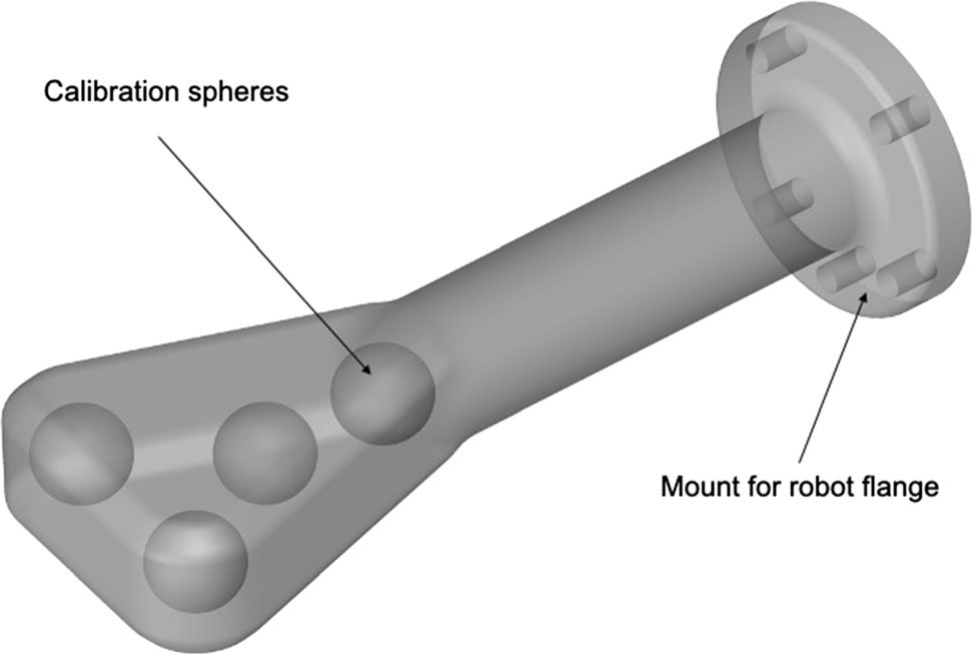
Semi-transparent CAD rendering of the calibration tool displaying the calibration spheres. The tool size is 200 × 75 × 30 mm

**Fig. 2:**
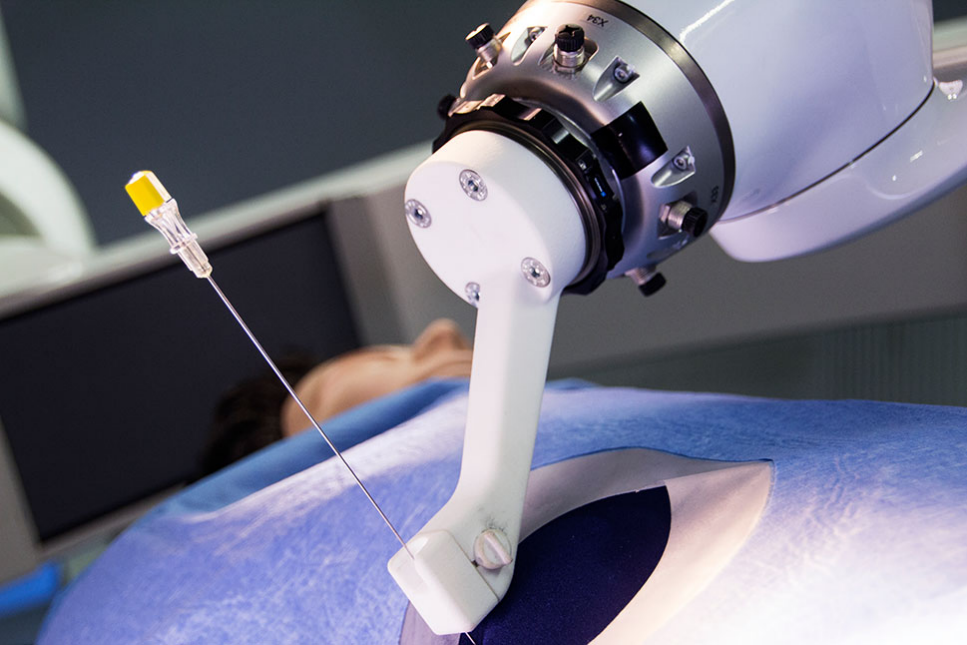
Guidance tool attached to the robotic arm with inserted needle

A lightweight robot (LBR iiwa 14 R820, KUKA AG, Germany) was mounted on a mobile base for fast setup, if needed, and to ensure that the system can be moved out of the immediate vicinity of the imaging system. The LWR features hand-guiding capabilities, translating forces exerted by the user into position, and hence, allowing for intuitive interaction with the RAS. To enable fast exchange of the robotic tools required for the setup, the robotic arm is equipped with a tool-changing system (FWS, SCHUNK GmbH & Co. KG, Germany) for manual tool exchange. Furthermore, the RAS can be equipped with a calibration or a guidance tool (prototype Fraunhofer IPA, Mannheim, Germany; Figs. 1 and 2). For robot-to-image calibration, the calibration tool is attached to the robot and placed inside the CBCT scan volume. The calibration tool was manufactured using a radiotranslucent plastic so as not to compromise image quality and contains four hollow spheres. These spheres, which are located at pairwise unique distances to each other inside the tool, can be automatically detected by the planning software in the CBCT imaging and thus enable robot-to-image calibration. Following calibration, the needle holder is attached to the robot in exchange for the calibration tool.

For imaging, a multi-axis C-arm CBCT system was used (Artis zeego, Siemens Healthineers, Germany) with a common OR table (Maquet GmbH, Germany). For needle placement, an abdominal phantom (Model 057A, CIRS.; Norfolk, USA), which contains multiple artificial target lesions was employed.

### Software

The inherent navigation software (prototype Fraunhofer MEVIS, Bremen, Germany) receives the DICOM images (Digital Imaging and Communications in Medicine) and communicates with the LWR both to calibrate the system and transfer the target. Software development was based on MeVisLab [10] and contains tools for both image segmentation [11] and image registration [12].

The graphical user interface (GUI) of the navigation software displays the state of the RAS as well as diagnostic, planning, and treatment data.

For communication between the navigation software and the RAS, an open-source protocol was employed for communication in systems for image-guided therapy [13].

### Interventional Workflow

**Fig. 3:**
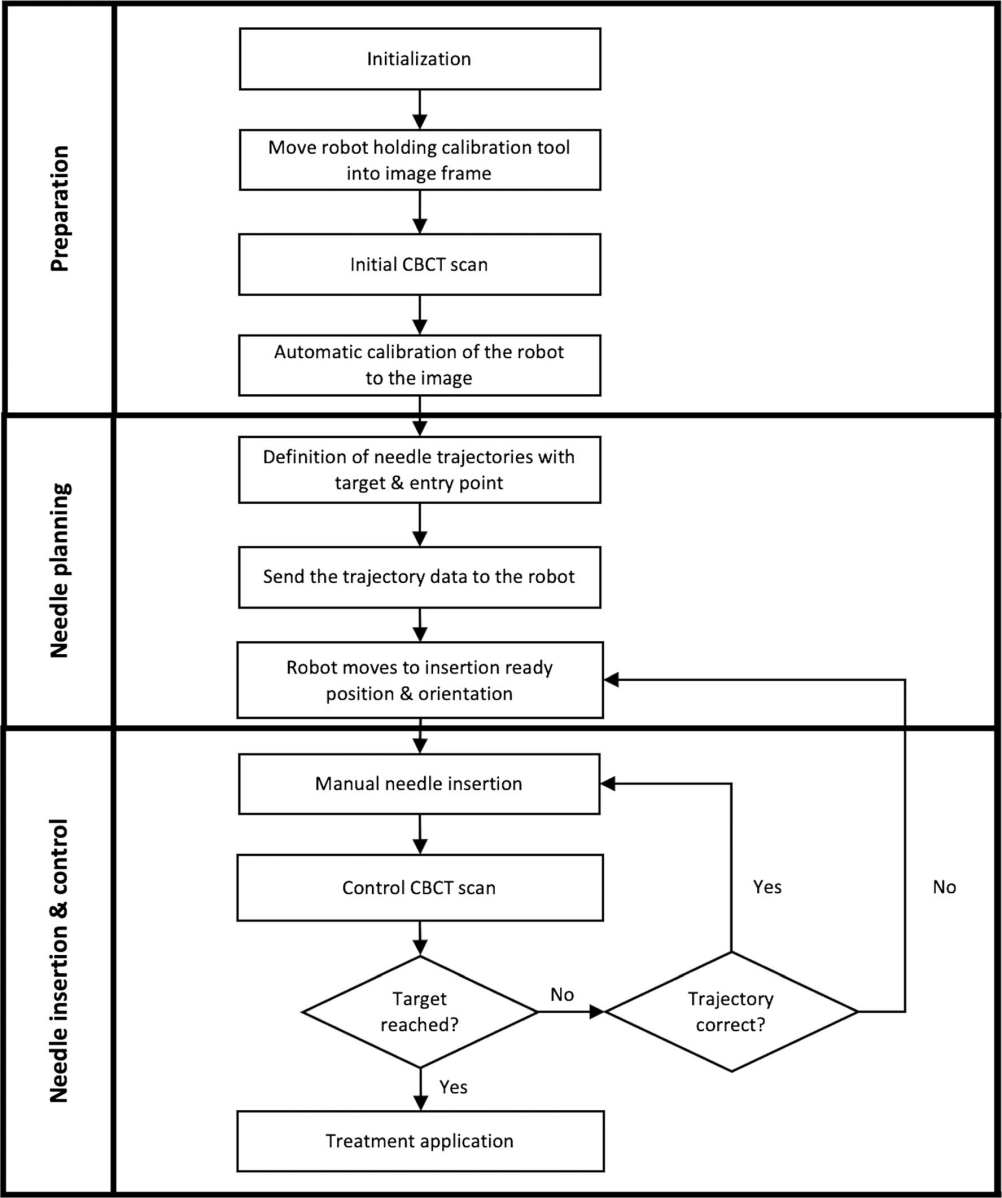
Workflow for robotic-assisted needle placement using the LWR in conjunction with a CBCT

The LWR on the mobile platform was positioned next to the table, opposite to the physician and as close to the imaging system as possible without colliding with the CBCT system. For calibration and initial planning of the needle path, a CBCT scan of the abdominal phantom was performed using the following parameters: 6 s, 200° rotation protocol with 0.5°/frame, generating a total of 397 projections. The assisted workflow is depicted in Fig. 3.

The needle trajectories were then planned by selecting the desired entry and target point in the imaging data of the phantom. This needle trajectory was then sent to the RAS. The LWR with the attached needle guidance tool, and then automatically moved into the position planned for the needle trajectory. Once the needle guide was in place, a 19G coaxial needle (TruGuide, C. R. Bard Inc.; Murray Hill, USA) was manually inserted. The insertion depth was known from the initial planning. After detaching the needle guidance tool and retracting the robotic arm, a control CBCT with the same settings as the planning CBCT was performed.

Eight lesions inside the abdominal phantom were targeted with one in-plane (RAO/LAO angulation) and one out-of-plane (craniocaudal angulation + RAO/LAO angulation) trajectory each, amounting to a total of 16 needle trajectories. The maximal possible angulations were 17.5° cranially, 32.5° caudally, and 20° RAO/LAO for the RAS.

**Fig. 4:**
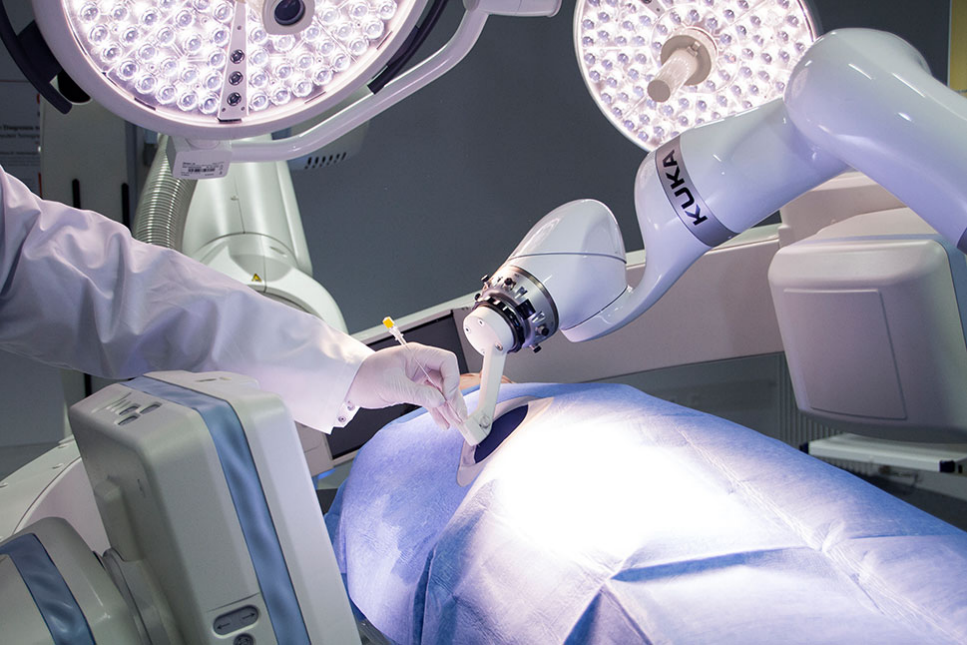
Experimental setup with the phantom placed on the operating room table: CBCT system with LWR and needle being advanced along the trajectory

After a brief introduction to the system, an experienced interventional radiologist (> 500 CT-guided interventions) performed the interventions according to the workflow steps depicted in Fig. 3. The CBCT setup with LWR is depicted in Fig. 4.

### Evaluation

**Fig. 5:**
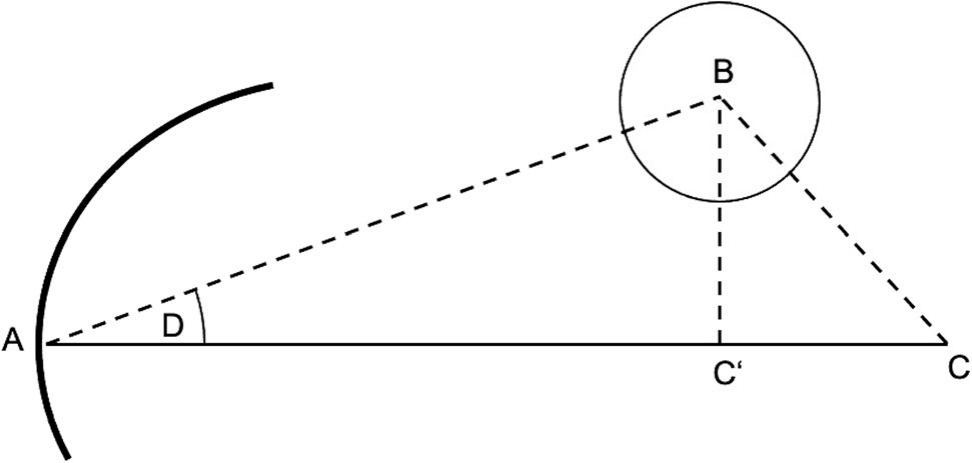
Schematic of the targeting error with entry point A, target point B, planned needle trajectory $\overrightarrow{\mathrm{AB}}$, actual needle tip C, actual needle $\overrightarrow{\mathrm{AC}}$, projected correct needle tip position C', and angulation error D

The interventions were recorded using four surveillance cameras in order to extract timing parameters for the aforementioned steps in this process. The targeting error consists of three different components, which are illustrated in Fig. 5. The experimental results show that the planned entry point (A) and the actual entry point can be considered identical based on the resolution of the control scan, which consisted of isotropic voxels with an edge length of 0.48 mm. The following possible deviations were evaluated: the actual needle tip (C) deviation from the planned target point (B), resulting in the absolute needle deviation; the angular deviation, which is the angle between the lines $\overrightarrow{AB}$ (planned needle trajectory) and $\overrightarrow{AC}$ (actual needle trajectory); and the longitudinal deviation as the distance from C' to C, which can occur since only the trajectory is determined by the RAS and the needle insertion depth is performed manually by the interventionist.

Time needed for needle placements was computed according to the timestamps of the CBCT scans and the recordings of the surveillance cameras. Procedural times were also recorded, whereby an intervention started with the planning scan and ended with the control scan.

### Statistical Analysis

Statistical analysis of the results and comparison to previous experimental results were performed using ANOVA: single-variant analysis of variance (Excel; Microsoft Corp, Redmont, Washington, USA).

For this phantom study Institutional Review Board (IRB) approval was not necessary, as no studies with human participants or animals were performed.

## Results

In all 16 interventions the planned target was hit, resulting in a 100% technical success rate. Individual results for targeting precision and number of needle position corrections (iterations) are displayed in Table 1. Overall, for in- and out-of-plane trajectories mean angular, absolute, and longitudinal deviation was 1.12°, 2.74 mm, and 2.14 mm, respectively. There was no need to correct any needle position (iterations). Overall mean procedural time, including the time required for calibration and control scan, was 361 s ± 43 s. Comparing results for in-plane and out-of-plane needle guidance, there was no statistically significant difference (*p* > 0.05) in precision. A slight statistically significant difference could be found (*p* = 0.0214) between the duration of in- and out-of-plane procedures (337 s vs. 380 s).

**Table 1:** The results of needle placement using CBCT with robotic assistance. Mean value, standard deviation (STD), minimum, and maximum

	Angular deviation D [°]	Absolute deviation $\left| {\overrightarrow {BC} } \right|$ [mm]	Longitudinal deviation $\left| {\overrightarrow {CC^{\prime}} } \right|$ [mm]	Iteration	Procedural time [sec]
Overall	1.12 STD = 0.68 0.4–2.7	2.74 s = 1.34 1.0–5.9	2.14 STD = 1.55 0.2–5.8	0	361 STD = 43 278–429
In-plane	1.06 STD = 0.61 0.5–2.81	2.84 STD = 1.65 1.0–5.9	2.25 STD = 1.82 0.2–5.8	0	337 STD = 50 305–497
Off-plane	1.19 STD = 0.74 0.4–2.7	2.62 STD = 0.85 0.4–2.6	2.01 STD = 1.16 0.5–3.6	0	380 STD = 26 332–429
*p*-value (in-/off-plane)	0.4841	0.8729	0.7428	–	0.0214

## Discussion

The advantages of an open, multi-axis C-arm system include improved access to the patient and a higher degree of flexibility to allow for out-of-plane needle trajectories since the image quality of CBCT is high enough for pre-, intra-, and post-procedural imaging [14]. The results of different studies can be difficult to compare since the approaches for determining interventional times or precision are different and standard CT was often used for image guidance. Guiu et al. evaluated a RAS in a preclinical living animal model using CT for imaging [15]. They entered 17G needles percutaneously to 5 mm targets, which had been previously installed in the liver. Their RAS also predefined the chosen trajectory and the needles were advanced manually. Possible needle tip deviations were evaluated. Of the needle insertions, correction was required in 19.4% because the needle tip deviated more than 5 mm from the target, and 76.6% of the needles showed a mean lateral deviation of 3.3 mm. Neither angulation or trajectory length nor the experience of the manipulator had any impact on the precision of the needle guidance in their study using a living animal model while puncturing the liver in a CT scanner. Comparing their results to those of this study, accuracy of the two RAS seem to be similar, although a phantom was employed in the present study.

In a previous experiment, our group compared an integrated 3D laser navigation tool of a multi-axis CBCT system for needle guidance to a MDCT integrated planning tool in terms of accuracy and time for needle placement [6]. The study results showed that the two guidance tools were precise and equal to one another in terms of accuracy. Comparing those results with the ones of this study, all three methods seem comparably accurate. The main difference can be found in the overall procedural duration: mean overall procedural time needed to place a needle in a target was 614 s with MDCT and 904 s with CBCT guidance. In this study, mean overall procedural time was 361 s, which is 1.5 and up to 2.5 times faster than for conventional, manual MDCT, and CBCT guidance. Additionally, there was a statistically significant difference between in- and out-of-plane guidance in this study favoring in-plane guidance. Comparing minimum to maximum procedural time, this slight difference of 43 s seems to be negligible, however, since the standard deviation of out-of-plane interventions is also half of the value of in-plane procedures (26 s vs. 50 s).

In terms of precision, no significant difference was observed between the aforementioned manual needle placement into a phantom using MDCT and CBCT guidance and the CBCT with robotic guidance conducted in this study. Mean angulation errors differ by around 1° and mean absolute deviations differ by a maximum of only around 1 mm.

In a study using CBCT-integrated laser guidance for marking intraoperative pulmonary lesions prior to resection, it took on average 35 min to place the guidewire [16]. Here, robotic assistance may potentially significantly reduce the time needed for guidewire placement. Further advantages may be a certain democratization of percutaneous interventions, allowing less experienced user to achieve precise and time-effective results when using robotic assistance. A disadvantage of using a LWR may be the additional space required for the mobile platform and the LWR itself, especially when compared to integrated guidance solutions such as CBCT laser guidance.

The main limitation of the current study was the phantom setting despite using a dedicated, commercially available biopsy phantom. Precision, possible time reduction, and safety still need to be proven in a preclinical setting. Additionally, tissue deformation and breathing motion could not be simulated using this phantom. A further limitation of the system presented here is the size of the field of view (FOV) that the CBCT system provides. For large patients, the calibration tool and the target lesion might not be depicted on the same scan volume. This problem can be addressed by using special imaging protocols for large volume scans or by newer generations of CBCT with larger FOVs. A possible 2D–2D calibration method might omit this problem. Imaging quality of CBCT scans is commonly significantly lower than MDCT systems. Although the current detector generation has already improved the contrast-to-noise ratio, soft-tissue imaging still is suboptimal. Image fusion with preinterventional imaging may resolve this issue and might also have further advantages (such as reducing planning time, less radiation exposure of patient and medical staff, higher precision, and lower complication rates).

## Conclusion

By using the RAS presented here for percutaneous, image-guided needle placement in combination with a multi-axis C-arm CBCT system, needle placement was precise and effective in this phantom setting.

## Declarations

### Employment

Andreas Rothfuss was employed with Fraunhofer IPA until 2020-03 and has hence joined BEC GmbH. BEC is driving the further development of the presented system and intents to bring it to market.
